# Antibiofilm activity of silver nanoparticles biosynthesized using viticultural waste

**DOI:** 10.1371/journal.pone.0272844

**Published:** 2022-08-10

**Authors:** Anna Miškovská, Michaela Rabochová, Jana Michailidu, Jan Masák, Alena Čejková, Jan Lorinčík, Olga Maťátková

**Affiliations:** 1 Department of Biotechnology, University of Chemistry and Technology, Prague, Czech Republic; 2 Research Centre Řež, Husinec, Czech Republic; 3 Faculty of Biomedical Engineering, Czech Technical University in Prague, Kladno, Czech Republic; King Abdulaziz University, SAUDI ARABIA

## Abstract

Green methods have become vital for sustainable development of the scientific and commercial sphere; however, they can bring new challenges, including the need for detailed characterization and elucidation of efficacy of their products. In this study, green method of silver nanoparticles (AgNPs) production was employed using an extract from grapevine canes. The aim of the study was to contribute to the knowledge about biosynthesized AgNPs by focusing on elucidation of their antifungal efficiency based on their size and/or hypothesized synergy with bioactive substances from *Vitis vinifera* cane extract. The antifungal activity of AgNPs capped and stabilized with bioactive compounds was tested against the opportunistic pathogenic yeast *Candida albicans*. Two dispersions of nanoparticles with different morphology (characterized by SEM-in-STEM, DLS, UV-Vis, XRD, and AAS) were prepared by modification of reaction conditions suitable for economical production and their long-term stability monitored for six months was confirmed. The aims of the study included the comparison of the antifungal effect against suspension cells and biofilm of small monodisperse AgNPs with narrow size distribution and large polydisperse AgNPs. The hypothesis of synergistic interaction of biologically active molecules from *V*. *vinifera* extracts and AgNPs against both cell forms were tested. The interactions of all AgNPs dispersions with the cell surface and changes in cell morphology were imaged using SEM. All variants of AgNPs dispersions were found to be active against suspension and biofilm cells of *C*. *albicans*; nevertheless, surprisingly, larger polydisperse AgNPs were found to be more effective. Synergistic action of nanoparticles with biologically active extract compounds was proven for biofilm cells (MBIC_80_ 20 mg/L of polydisperse AgNPs in extract), while isolated nanoparticles suspended in water were more active against suspension cells (MIC 20 mg/L of polydisperse AgNPs dispersed in water). Our results bring new insight into the economical production of AgNPs with defined characteristics, which were proven to target a specific mode of growth of significant pathogen *C*. *albicans*.

## Introduction

The green approach to the synthesis of metal nanoparticles (NPs) is a current topic in modern nanobiotechnology. It responds to the need to develop sustainable methods for the synthesis and production of nanomaterials on an industrial scale. Unlike traditional methods of NPs preparation (chemical and physical), green methods mediate the synthesis of NPs using compounds of natural origin, such as proteins, alkaloids, flavonoids, reducing carbohydrates, polyphenols, and others, which act as both reducing and stabilizing agents [1]. In particular, whole living plants, plant extracts (aqueous, ethanolic, methanolic), microbial cells, cell-free extracts of microorganisms (supernatants, filtrates), or, for example, residual media after cell cultivation are most often the source of these biomolecules. Green methods are generally environmentally friendly, do not use organic solvents or toxic substances, and are inexpensive [2, 3]. Although green synthesis undoubtedly reduces the environmental impact of NPs production, the rational use, recycling and disposal of NPs is also important [4].

Phytosynthesis using plant extracts is gaining more and more attention, thanks to the many advantages of this procedure, including a wide variety of materials that could be used for extract preparation, simplicity of the whole process, use of the gentle reaction conditions (atmospheric pressure, room temperature, gentle pH values) and easy scale-up of the whole process [4]. Extracts of various plants (*Hibiscus rosa sinensis*, *Eucalyptus globulus*, *Azadirachta indica* or black tea), plant parts (root, leaf, fruit, stem, flower, bark, and bud) or waste after industrial processing of plants (*Brassica oleracea* waste leaves) could be used in plant-extract mediated synthesis of silver nanoparticles (AgNPs) [5–7]. The use of complex plant extracts leads to the rapid synthesis (even in a few minutes) of biocompatible and very stable nanoparticles. Another advantage of using plant extracts is the flexibility of reaction conditions setting, which is broader compared to microbial biosynthesis, where manipulation, e.g. with reaction temperature or pH, is considerably limited due to the presence of microorganisms [8]. The reaction conditions are very important in terms of the desired properties of NPs [9]. By their manipulation, the physicochemical properties of NPs, such as size, polydispersity, shape, and surface functionalization, could be significantly affected. For example, the pH of the reaction mixture is an important parameter in the biosynthesis of NPs mediated by plant extracts and many studies have confirmed that it affects the reaction rate, morphology and stability of the resulting nanoparticles [10]. This is due to the increased formation of nucleation centers along with an increase in pH. At the same time, pH affects the activity of biomolecules involved in the reduction and stabilization of nanoparticles [11].

It has already been found that the physicochemical properties of NPs significantly affect their antimicrobial activity [9, 12]. This is particularly interesting in connection with AgNPs, which are known for their antimicrobial effects. Among the main advantages of using silver nanoparticles over conventional antimicrobials is mainly the low risk of development of resistance, due to the wide range of mechanisms of NPs action [13]. The excellent antimicrobial activity of AgNPs has already been described against many pathogenic bacteria (gram positive *Staphylococcus aureus*, *Bacillus cereus*, and gram negative *Escherichia coli*, *Pseudomonas aeruginosa*, *Salmonella choleraesuis*), yeasts (*Candida* sp.) and fungi (*Aspergillus* sp., *Penicillium* sp. *Fusarium* sp., *Pythium* sp.) [14–17]. The attractiveness of biogenic NPs lies in their functionalization, capping and stabilization by biomolecules, which themselves might possess antimicrobial properties and also allow NPs to bind to the cell surface [12]. Since the extracts are prepared exclusively from non-toxic plants and often have beneficial effects on their own, the use of a combination of two active substances (NPs and plant extract) is an interesting approach. The combination could have synergistic effects and thus allow to reduce the effective concentrations of the individual agents, as described, for example, in a study where AgNPs with essential oils were applied against several pathogenic bacterial species and fungi and significant synergistic inhibitory effects were observed [18]. The potential of biosynthesized nanoparticles in medicine is aimed mostly at applications, where they could present a defence barrier to prevent infection. Specifically, they could replace traditionally prepared AgNPs in applications such as wound dressings or AgNPs-coated implants.

The present study deals with the green synthesis of AgNPs using viticultural waste, *Vitis vinifera* canes. Most current studies focused on the synthesis of silver nanoparticles by *V*. *vinifera* use whole fruits or fruit skin extracts of this plant [19–21]. In contrast, this study focuses on the use of canes as they are a waste product of vine growing without other applied use and represent a stable (in storage) and very rich source of bioactive substances that can contribute to the synthesis of stable nanoparticles of various properties, as demonstrated by our results. By adjustment of the reaction conditions, AgNPs of different size and polydispersity were biosynthesized, and their antimicrobial and antibiofilm activity against the opportunistic pathogenic yeast *Candida albicans* was studied. The aim was to describe the antifungal effects of AgNPs of different physicochemical properties (size and polydispersity) and simultaneously investigate whether the combination of the extract remaining after synthesis in the NPs colloid will improve the resulting antimicrobial effect. In addition, the interaction of nanoparticles with the surface of treated cells was studied by SEM imaging, to better understand the mechanism of NPs action.

## Materials and methods

### Plant material and preparation of extract

Grape canes from both white and red varieties of *Vitis vinifera* (Pinot gris, Pinot noir and Rheinriesling) were collected from Grébovka vineyard (Prague, Czech Republic, wine region Bohemia) during the dormancy period in January 2017. The extraction method was modified according to Rollová, Gharwalova [22]. Plant material was dried and crushed using high-speed mixer. To prepare the ethanolic extract of *Vitis vinifera* canes, the obtained powder was mixed with 40% (v/v) ethanol (1:4 (w/v)) (Penta, Czech Republic). The mixture was extracted for 24 hours at room temperature in the dark. After that, the extract was filtered through a laboratory filter and subsequently through a microfilter with porosity 0.22 μm. For further use, the liquid extract had been stored at 4 °C in the dark.

### Synthesis of AgNPs and their isolation

AgNPs were synthesized combining the ethanolic extract of *V*. *vinifera* canes (obtained as described above) with AgNO3 (final concentration 1 mM, purchased from VWR, USA).

The AgNPs with different morphology were prepared under distinct reaction conditions, that were selected based on initial experiments where different AgNO_3_ molarities, extract volume, order of reagent addition, pH, and light exposure were studied with the aim of identifying key variables influencing the NPs morphology. To prepare monodisperse nanoparticles (mAgNPs), 8 ml of the extract was added to a 72 ml of AgNO_3_ aqueous solution (distilled water) to obtain final concentration 1 mM. The pH of obtained mixture was immediately adjusted to 7.5 by adding 1% (w/v) NaOH (Sigma-Aldrich, USA) and the mixture was placed on an orbital shaker (150 rpm) in the dark for 72 hours. To prepare polydisperse nanoparticles (pAgNPs), 4 ml of ethanolic *V*. *vinifera* extract was mixed with 36 ml of AgNO_3_ (final concentration 1 mM) and the mixture was left in daylight for 12 hours in 100 ml Erlenmeyer flask and then was placed in the dark to another 60 hours (without stirring). Additionally, biosynthesized NPs were isolated into ultrapure water to allow for a comparison of NPs antimicrobial activity with the *Vitis vinifera* extract remaining from the reaction (mAgNPs/e, pAgNPs/e) and isolated nanoparticles (mAgNPs/w, pAgNPs/w). For that, biosynthesized nanoparticles were isolated from the extract by centrifugation at 49,054 × g for 30 min (Sorvall Evolution RC, Kendro, Denmark), and the pellet of nanoparticles was resuspended in ultrapure water (Type 1, obtained by Direct-Q^®^ Water Purification System, Merck, Germany). Isolation of the NPs was verified by UV-Vis spectra analysis of the resulting supernatant. For further use, nanoparticles were stored at 4 °C in the dark.

### Characterization of AgNPs by UV-Vis

The formation of AgNPs was at first indicated by color change of the solutions and confirmed by UV-Vis spectrophotometry. The UV-Vis absorption spectra in the range 300–700 nm were analyzed using a Reader Infinite M900Pro (TECAN MTP, Switzerland). UV-Vis spectroscopy was also used for monitoring the stability of AgNPs for 6 months. For this purpose, nanoparticles were stored in the dark at 4 °C and their spectra were monitored in time intervals: 1 week; 1, 2, 4, and 6 months.

### Characterization of AgNPs by STEM-in-SEM

The imaging of the synthesized NPs was performed using a scanning transmission electron microscopy (STEM) detector integrated in a Focused Ion Beam–Scanning Electron Microscope (FIB-SEM) instrument LYRA3 (TESCAN ORSAY HOLDING, a.s., Czech Republic). The samples were analyzed at an electron beam energy of 30 keV with the STEM detector in a bright field (BF) mode. The BF mode provided high contrast images of nanoparticles, which were used for the determination of nanoparticle morphology (particle size and shape distributions).

Image analysis was performed in ImageJ [23]. Every image was calibrated prior to image analysis operations. The primary parameters obtained for every particle from the image processing were area ***A*** in units of nm^2^ and perimeter ***P*** in units of nm. Particle area ***A*** is defined as the projection of a particle shape onto a plane of the electron beam in focus. Based on those two parameters, equivalent circular area diameter ***d***_***ECD***_ and circularity ***f***_***CIRC***_ of every particle were calculated. The ***d***_***ECD***_ is defined as the diameter of a circle that has the same area as that of a particle, $d_{ECD}=2∙\sqrt{A/\pi }$. The ***f***_***CIRC***_ is defined as the ratio of the area of a particle to the area of a circle with the same perimeter, *f*_*CIRC*_ = 4 ⋅ *π* ⋅ *A*/*P*^2^. The circularity value of a perfect circle is 1.0, lower circularity value indicates larger deviation of a given particle from a perfect circle. Subsequently, histograms of the size ***d***_***ECD***_ and shape ***f***_***CIRC***_ distribution of biosynthesized nanoparticles using *V*. *vinifera* cane extract were prepared. Average values and standard deviations were calculated. The counts (n) of analyzed nanoparticles were as follows: n_(mAgNPs/e)_ = 1157; n_(mAgNPs/w)_ = 480; n_(pAgNPs/e)_ = 708; n_(pAgNPs/w)_ = 558.

### Characterization of AgNPs by DLS

The hydrodynamic diameter together with zeta potential were determined using dynamic light scattering (DLS). DLS analysis was performed using Zetasizer Pro (Malvern Panalytical, United Kingdom). The data were processed using ZS Xplorer software v1.50 (Malvern Panalytical, United Kingdom).

### Characterization of AgNPs by XRD

The crystalline structure of biosynthesized AgNPs was investigated by X-Ray Diffraction (XRD) crystallography using PANanalytical X´Pert PRO diffractometer (PANanalytical, Netherlands) with a CuKα radiation (generator operations U = 40 kV, I = 30 mA). For this purpose, nanoparticles were washed three times in ultrapure water and centrifuged, the obtained pellet of NPs was lyophilized using Heteo PowerDry LL 3000 Freeze Dryer (Thermo Electron Corporation, USA).

### Determination of mass concentration of AgNPs by AAS

The mass of silver in the particles was determined using atomic absorption spectrometer Agilent 280FS AA (Agilent, USA). For that, nanoparticles were first isolated by centrifugation (30 min, 49,054 × g). Supernatant was used for AAS analysis, and the mass concentration together with the yield of the synthesis were calculated from these results using the known initial concentration of silver ions at the start of the synthesis.

### Microorganisms, growth media and conditions

Model clinical isolate strain *Candida albicans* ATCC 10231 originated from the American Type Culture Collection (ATCC). The inoculum was pre-cultured before each experiment by adding the cryopreserved microorganism (stored at -70 °C) to a fresh yeast extract peptone dextrose broth medium (YPD–Yeast extract 10 g/L; Peptone 20 g/L; Dextrose 20 g/L; all Sigma-Aldrich, USA) and incubating for 24 h, at 37 °C, 150 rpm in an orbital shaker. The antifungal experiments were carried out in Roswell Park Memorial Institute 1640 medium (RPMI-1640) with L-glutamine, without sodium bicarbonate (Sigma-Aldrich, USA).

### Antifungal activity of AgNPs against planktonic cells

The activity of AgNPs against planktonic cells was determined by the microdilution method. The cultivation of yeasts was carried out in microtiter plates using Bioscreen C analyzer (Oy Growth Curves Ab Ltd., Finland). AgNPs were added in concentration range 2.5–40.0 mg/L (2.5, 5.0, 10.0, 20.0, 40.0 mg/L; dilution in phosphate buffered saline solution, PBS, pH = 7.4). Aliquots of 162 μl of fresh RPMI-1640 medium (used for the antifungal susceptibility testing of *Candida* sp. according to CLSI) were added into each well. Finally, wells were filled up to a final volume 320 μl by adding 30 μl of standard yeast inoculum (OD_600 nm_ = 0.100 ± 0.010) prepared to a fresh RPMI-1640 medium, followed by incubation for 24 h at 37 °C. Nanoparticles-free control was filled with 130 μl of PBS, 162 μl of fresh RPMI-1640 medium and 30 μl of standard yeast inoculum. The activity of *V*. *vinifera* extract alone (in 10% aliquots of corresponding nanoparticle volume) was also monitored. Each experiment was performed in three independent replicates, 10 parallels each. Minimum concentration required to inhibit 80% of cell growth (MIC_80_) and 100% of cell growth (MIC) was determined as the lowest concentration that causes at least 80% resp. 100% decrease in growth after a 24 h incubation, according to the definition by Serra, Hidalgo-Bastida [24].

### SEM visualization of *Candida albicans* planktonic cells after treatment with AgNPs

Samples were gathered from control (nanoparticles-free) and treated cell cultures. The cell suspension was centrifuged (10 min, 9,000 × g) and washed twice with sterile saline solution, once with distilled water. Subsequently, an aliquot of 10 μl of washed cell culture (OD_600 nm_ = 0.3 ± 0.050) was dropped at silicon wafer (Sil’tronix Silicon Technologies, France) and samples were air-dried. The LYRA3 microscope (TESCAN ORSAY HOLDING, a.s., Czech Republic) was used for SEM imaging at an electron beam voltage of 10 kV.

### Antibiofilm activity of AgNPs

The pre-cultured inoculum (see section Microorganisms, growth media and conditions) was centrifuged (10 min, 9,000 × g) and resuspended in fresh RPMI-1640 medium. The biofilm experiments were carried out in 96-well polystyrene microtiter plates (TPP, Sigma-Aldrich, USA). Various concentrations of AgNPs ranging from 2.5–20 mg/L (two-fold dilution in PBS) were added to wells. Each well was topped up to 280 μl of final volume with 210 μl of standard yeast suspension (OD_600 nm_ = 0.800 ± 0.010) and incubated at orbital shaker for 24 h, at 37 °C. Afterwards, the wells were washed twice with sterile saline to remove non-adhered cells. The metabolic activity of adhered cells was observed using resazurin viability assay according to Vaňková, Kašparová [25]. An aliquot of 25 μl of resazurin (Sigma-Aldrich, USA) stock solution (0.15 g/L in sterile PBS), 25 μl of glucose (Penta, Czech Republic) (180 g/L in sterile PBS) together with 100 μl of sterile saline were added to each well and the plate was incubated for 30 min (at 37 °C in darkness). After incubation, the fluorescence intensity of resorufin was determined using Reader Infinite M900Pro at excitation and emission wavelengths 545 resp. 575 nm. Nanoparticles-free control was filled with 70 μl of PBS, and 210 μl of standard yeast inoculum. Each experiment was performed in 8 parallels in three independent replicates. The activity of *V*. *vinifera* extract on metabolic activity of the biofilm cells (in 10% aliquots of pipetted nanoparticle volume) was also monitored. The resulting effect of AgNPs on biofilm is expressed as % of the metabolic activity of the cells relative to the nanoparticle-free control, which was assigned to be 100%.

### Statistical analysis

For the evaluation of microbial growth (see section Antifungal activity of AgNPs against planktonic cells) or biofilm assays (see section Antibiofilm activity of AgNPs) data, we used Dixon’s Q test to exclude outlying values. Arithmetic means and standard deviations (SD) were calculated for each experiment and all results were expressed in relative percentages (control samples were 100%).

## Results and discussion

The aim of our work was to identify and implement conditions enabling synthesis of AgNPs of different morphology and size distribution with different properties, which would also enable easy large-scale production. The properties studied included the comparison of their biological activity against opportunistic pathogenic yeast *C*. *albicans*. Size and circularity of biosynthesized AgNPs was determined by analysis of STEM-in-SEM images and the NPs were further characterized by UV-Vis, DLS and XRD. In addition to studying the effect of size and polydispersity on antifungal and antibiofilm activities of NPs, the effect of dispersing medium (ethanolic extract of *V*. *vinifera* / ultrapure water) was also studied to reveal possible synergistic activity of AgNPs and plant extract. Therefore, four NPs dispersions were obtained and studied: monodisperse AgNPs with *V*. *vinifera* extract remaining from the reaction (mAgNPs/e), isolated monodisperse AgNPs resuspended into ultrapure water (mAgNPs/w), polydisperse AgNPs with *V*. *vinifera* extract remaining from the reaction (pAgNPs/e), and polydisperse AgNPs resuspended into ultrapure water (pAgNPs/w).

### Synthesis and characterization of AgNPs by UV-Vis

The biosynthesis of the AgNPs of different morphology was achieved by their formation under different reaction conditions using extract of *V*. *vinifera* canes with silver nitrate. Varied reaction conditions were tested (change of pH, temperature, exposure to light) maintaining constant input concentrations of the extract and silver nitrate. Two procedures were then selected from a set of experiments with regard to the size distribution and morphology of the resulting nanoparticles as well as the effectiveness of the whole synthesis with respect to potential scale-up and large-scale technology requirements. The aim was to synthesize NPs of different size distribution–monodisperse AgNPs (mAgNPs) and polydisperse AgNPs (pAgNPs).

To allow for a comparison of NPs antimicrobial activity with the *Vitis vinifera* extract remaining from the reaction, second set of NPs dispersions was obtained by including an isolation step. Isolation of the NPs was verified by UV-Vis spectra analysis of the resulting supernatant (see S1 Fig). Therefore, four NPs dispersions were obtained and studied mAgNPs/e, mAgNPs/w, pAgNPs/e, and pAgNPs/w.

**Fig 1:**
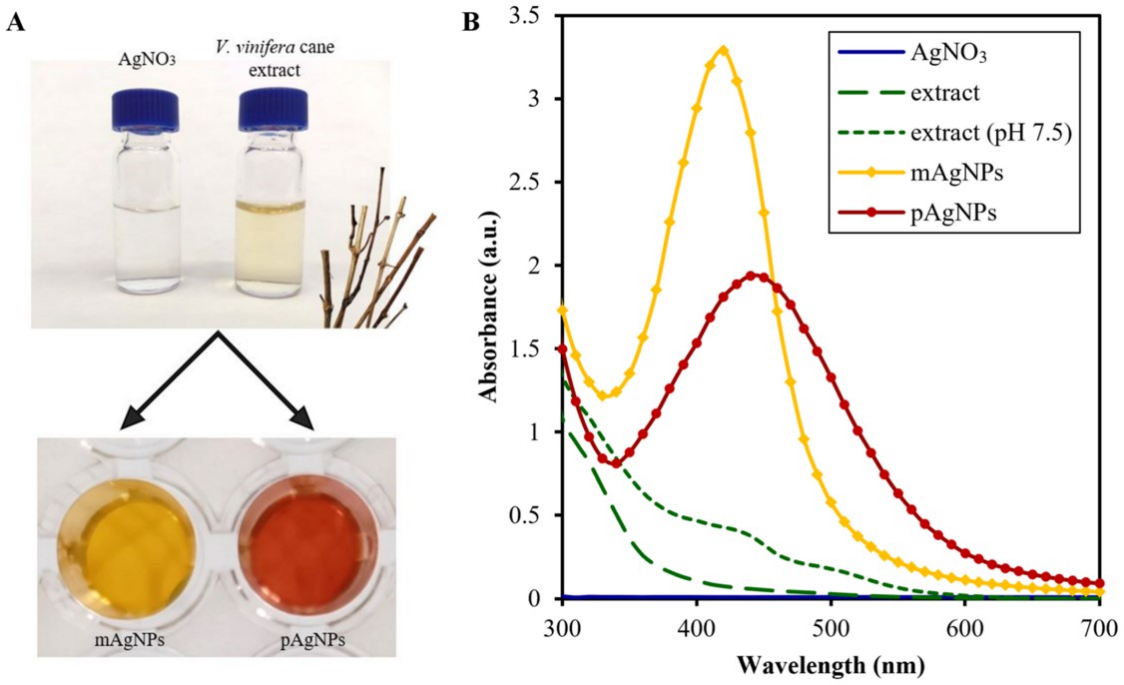
Scheme of AgNPs biosynthesis using *V*. *vinifera* extract and UV-Vis absorption spectra of prepared AgNPs. A–The colour of individual solutions–silver nitrate, *Vitis vinifera* cane extract, biologically synthesized monodisperse AgNPs (mAgNPs), biologically synthesized polydisperse AgNPs (pAgNPs); B–UV-Vis absorption spectra of controls and AgNPs.

The formation of nanoparticles was indicated primarily visually by colour change which occurs when nanoparticles are formed [26]. This change occurred immediately after reaction beginning in monodisperse AgNPs, resp. after 4 hours in polydisperse AgNPs. The colour of monodisperse nanoparticles changed from the original light brown to deep yellow, in contrast, the final colour of polydisperse AgNPs was dark red orange (Fig 1A). Both these colours are typical for silver colloids and occur due to the surface plasmon resonance (SPR). While yellow colour is characteristic for small spherical nanoparticles, the orange and red are typical for larger particles [27]. These conclusions were supported by follow-up characterization techniques such as UV-Vis, STEM-in-SEM and DLS of the NPs.

The results of UV-Vis analysis are shown in Fig 1B. The observed absorbance band at 340–600 nm is typical for the formation of AgNPs [28]. UV-Vis spectroscopy, in addition to confirming the formation of nanoparticles, also provides information about the size of AgNPs. The wavelength corresponding to the absorbance peak (λ_max_) reflects the size of the NPs, typically, small particles exhibit unique absorbance peaks at lower wavelengths and large vice versa [29]. The wavelengths corresponding to the peaks of the absorption bands were 420 nm for monodisperse AgNPs and 440 nm for polydisperse AgNPs, which is in agreement with following methods confirming that the mAgNPs were smaller than the pAgNPs (see sections Characterization of biosynthesized AgNPs by DLS and Characterization of biosynthesized AgNPs by STEM-in-SEM).

### Characterization of biosynthesized AgNPs by DLS

DLS measurements were employed to observe the hydrodynamic diameter and zeta potential (ζ) of the synthesized NPs (Table 1). The DLS data confirmed the successful biosynthesis of silver NPs of two different sizes–smaller mAgNPs with Z-average ~35 nm and bigger pAgNPs with Z-average ~102 nm. Hydrodynamic diameter was measured in both NPs–NPs in extract remaining after synthesis (AgNPs/e), and NPs isolated in ultrapure water (AgNPs/w), and it was confirmed that the hydrodynamic diameter did not change significantly after isolation. The measured zeta potential of mAgNPs was lower than –25 mV, which indicates high stability of the colloid [30]. Zeta potential of pAgNPs was higher than ζ of mAgNPs, however subsequent stability monitoring with UV-Vis did not confirm that either pAgNPs or mAgNPs aggregated in a time period of 6 months (see section Monitoring the stability of AgNPs over time using UV-Vis). The negative surface charge of the obtained AgNPs is attributed to the biomolecules present in the *V*. *vinifera* cane extract, which adsorbed to the NPs surface and stabilized the NPs.

**Table 1:** DLS of biosynthesized AgNPs. (mAgNPs–monodisperse AgNPs); pAgNPs–polydisperse AgNPs; e–*V*. *vinifera* extract; w–ultrapure water as medium).

Type of NPs	Z-average (nm)	Zeta potential (mV)
mAgNPs/e	34.43 ± 1.37	-30.04 ± 2.37
mAgNPs/w	35.33 ± 3.83	-25.14 ± 4.56
pAgNPs/e	101.63 ± 3.92	-21.24 ± 5.38
pAgNPs/w	103.35 ± 4.89	-24.53 ± 0.90

### Characterization of biosynthesized AgNPs by STEM-in-SEM

**Fig 2:**
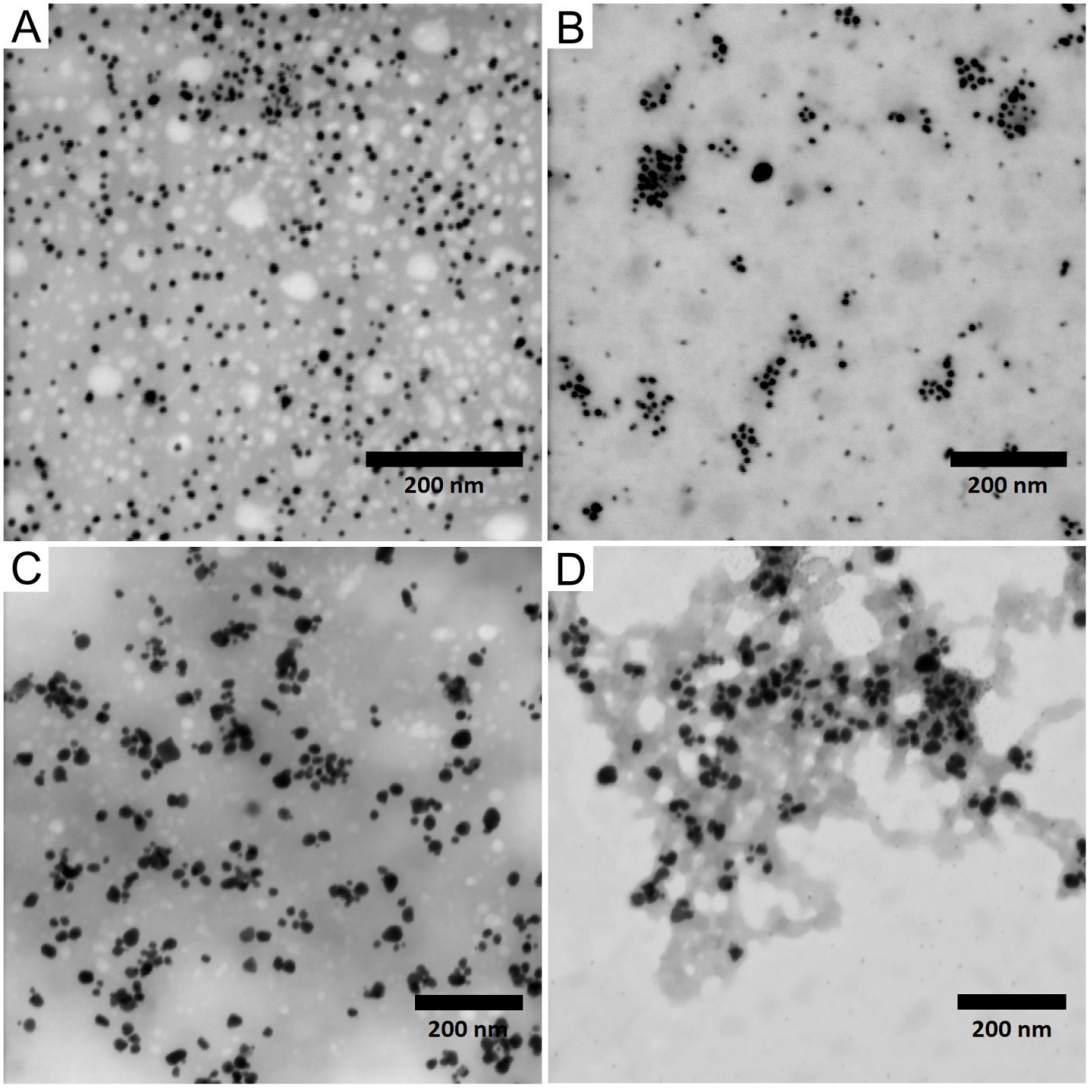
STEM-in-SEM images of biosynthesized nanoparticles. A–monodisperse AgNPs in extract as medium; B–monodisperse AgNPs in water as medium; C–polydisperse AgNPs in extract as medium; D–polydisperse AgNPs in water as medium; scale = 200 nm.

The imaging of biosynthesized NPs was performed using STEM-in-SEM. Fig 2 shows all samples of AgNPs. Monodisperse NPs (Fig 2A and 2B) were confirmed to contain smaller particles without aggregates with a narrow size distribution. The polydisperse NPs were found to be more diverse in size and shape (Fig 2C and 2D).

Detailed characterization of NPs was done using images from STEM-in-SEM. Using image analysis, the diameter and circularity of nanoparticles were determined, and subsequently analyzed as the distribution of nanoparticles in circularity intervals.

The average diameter and circularity of biosynthesized silver NPs are listed in Tables 2 and 3. The results of the image analysis are consistent with the findings from the UV-Vis and DLS measurements. The mAgNPs contained small particles with a narrow size range and uniform in shape (almost 90% of mAgNPs lies in the circularity interval 0.85–1.0). On the other hand, pAgNPs were larger, and 30% of the pAgNPs fell into a circularity range lower than 0.85 (corresponding to various shapes, distinct from circular form). The results shown in Table 2 also provide information about the diameter and shape stability of AgNPs after isolation into ultrapure water, when both parameters remain unchanged. The high stability of our biosynthesized AgNPs after resuspension in ultrapure water is consistent with previous studies that confirm that water is an ideal medium for the long-term storing of AgNPs [31].

**Table 2:** The average diameter and circularity, including standard deviation, of biosynthesized AgNPs. (mAgNPs–monodisperse AgNPs; pAgNPs–polydisperse AgNPs; /e–*V*. *vinifera* extract; /w–ultrapure water as medium).

Type of NPs	Average diameter of NPs (nm)	Average circularity of NPs (-)
mAgNPs/e	10.6 ± 2.7	0.92 ± 0.06
mAgNPs/w	10.8 ± 3.4	0.93 ± 0.06
pAgNPs/e	17.2 ± 6.9	0.89 ± 0.09
pAgNPs/w	18.3 ± 6.9	0.87 ± 0.09

**Table 3:** Percentage of biosynthesized AgNPs in circularity intervals (mAgNPs–monodisperse AgNPs; pAgNPs–polydisperse AgNPs; /e–*V*. *vinifera* extract; /w–ultrapure water as medium).

	Percentage of NPs with the bin (%)			
Circularity interval (-)	mAgNPs/e	mAgNPs/w	pAgNPs/e	pAgNPs/w
⟨0.00; 0.70)	0.9	0.4	3.4	5.2
⟨0.70; 0.75)	1.4	1.9	3.4	3.4
⟨0.75; 0.80)	2.9	2.9	7.1	9.3
⟨0.80; 0.85)	5.9	6.5	12.9	16.1
⟨0.85; 0.90)	17.9	17.1	22.6	26.9
⟨0.90; 0.95)	44.9	30.8	28.7	24.0
⟨0.95; 1.00⟩	26.2	40.4	22.0	15.1

AgNPs have been stored in the form of a colloidal solution that does not tend to separate because the submicron particles sediment so slowly that the effect is eliminated by the mixing tendencies of diffusion and convection [32]. The liquid storage is mostly preferred upon dry storage of AgNPs, that is usually difficult to disperse.

**Fig 3:**
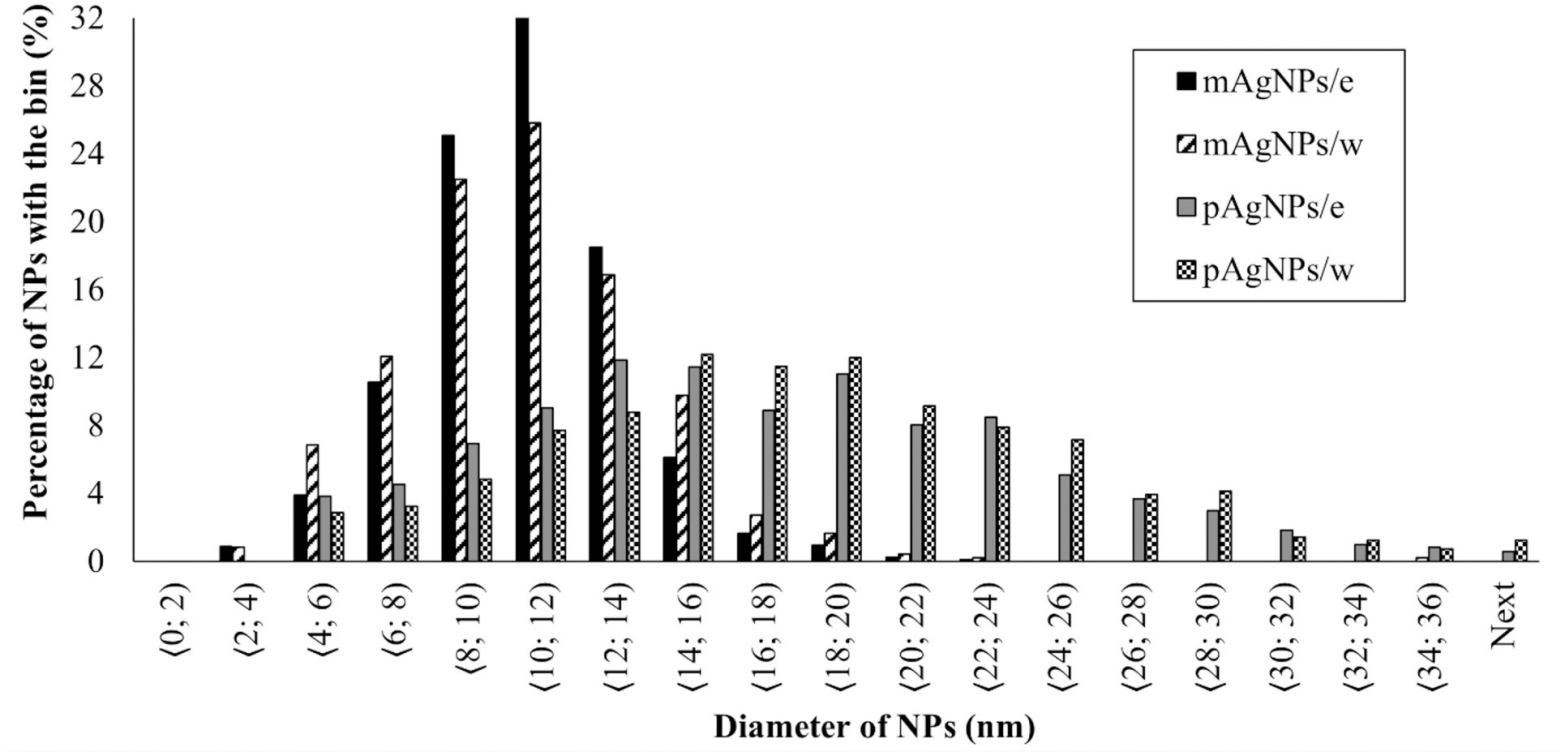
Size distribution of AgNPs biosynthesized using *V*. *vinifera* extract. (mAgNPs–monodisperse AgNPs; pAgNPs–polydisperse AgNPs; /e–*V*. *vinifera* extract; /w–ultrapure water as medium).

Subsequently, histograms of the particle size distribution of biosynthesized nanoparticles using *V*. *vinifera* cane extract are shown in Fig 3.

### Characterization of silver NPs by XRD

The crystalline structure of AgNPs was confirmed by X-Ray Diffraction (XRD) crystallography. The presence of face-centered cubic (fcc) structure of metallic silver was confirmed in both, mAgNPs and pAgNPs, by finding the characteristic diffraction peaks at 2θ values of 38.1114, 44.1933, 64.5215, 77.4042, 81.5175 (for mAgNPs), and 38.1166, 44.2196, 64.4865, 77.4269, 81.4545 (for pAgNPs) indexed to Bragg reflections of the (111), (200), (220), (311), (222) planes.

### Determination of mass concentration of AgNPs by AAS

The reaction yield and the determination of the Ag concentration present in the form of nanoparticles was conducted by AAS. While the initial concentration of silver ions in both reaction mixtures was identic, as expected, the yield of the individual methods of synthesis differed. The yield of the reaction leading to the formation of monodisperse AgNPs was very high (98%) and the concentration of silver in NPs was found to be 105.4 ± 0.1 mg/L. In comparison, the yield of the reaction leading to the formation of polydisperse nanoparticles was lower (91%) and the concentration was found to be 97.9 ± 7.2 mg/L.

### Monitoring the stability of AgNPs over time using UV-Vis

Apart from the characterization of the AgNPs, UV-Vis spectroscopy is commonly used to study the stability of AgNPs over time. In unstable colloids, the plasmon resonance peak shifts to longer wavelengths and broadens due to aggregation of NPs [33]. By monitoring the stability of the prepared nanoparticles, it was found that the wavelength corresponding to the absorbance peak (λ_max_) of all AgNPs remain unchanged in the observed time period (6 months). The results indicate high stability with no aggregation of both, mAgNPs and pAgNPs, regardless of the dispersing medium (*V*. *vinifera* extract/ultrapure water) (Table 4).

**Table 4:** Monitoring of stability of AgNPs synthesized using extract from *Vitis vinifera* canes. Monitored by UV-Vis (storage conditions: 4 °C, dark), (mAgNPs–monodisperse AgNPs; pAgNPs–polydisperse AgNPs; /e–*V*. *vinifera* extract; /w–ultrapure water as medium).

Type of NPs	Time	λ_max_ (nm)	A_max_ (-)
mAgNPs/e	1 week	420	3.10 ± 0.05
	1 month	420	3.32 ± 0.07
	2 months	420	3.38 ± 0.04
	4 months	420	3.41 ± 0.08
	6 months	420	3.30 ± 0.02
mAgNPs/w	1 week	420	3.07 ± 0.07
	1 month	420	3.10 ± 0.08
	2 months	420	3.09 ± 0.09
	4 months	420	3.12 ± 0.05
	6 months	420	2.97 ± 0.03
pAgNPs/e	1 week	440	1.84 ± 0.09
	1 month	440	2.01 ± 0.02
	2 months	440	2.03 ± 0.07
	4 months	440	2.14 ± 0.03
	6 months	440	2.18 ± 0.13
pAgNPs/w	1 week	440	1.77 ± 0.07
	1 month	440	1.92 ± 0.06
	2 months	440	2.03 ± 0.08
	4 months	440	1.94 ± 0.09
	6 months	440	1.96 ± 0.11

### Antifungal activity of AgNPs against planktonic cells of *Candida albicans* and SEM visualization cell-AgNPs interactions

AgNPs exhibit promising antimicrobial activity against many human pathogens (eg. *C*. *albicans*, *C*. *parapsilosis*, and *C*. *glabrata*), making them attractive to several fields, such as biomedicine, textile industry, food packaging or cosmetics [34, 35]. The antimicrobial effects of nanoparticles are influenced by their physicochemical properties, such as their size, shape, specific surface area, surface energy, charge, zeta potential, surface morphology, crystal structure or ligands [12]. Therefore, we have decided to study the antifungal activity of two different biosynthesized AgNPs dispersions–small monodisperse AgNPs (mAgNPs) and larger polydisperse AgNPs (pAgNPs), both prepared using *Vitis vinifera* cane extract. Moreover, the possibility of exploiting the synergistic activity of the plant extract (resulting from the biosynthesis process) and NPs was studied. For this purpose, AgNPs were isolated from original colloid by centrifugation and the pellet was resuspended into ultrapure water. The activity of AgNPs in *V*. *vinifera* extract remaining after synthesis (AgNPs/e) as well as the activity of isolated nanoparticles (AgNPs/w) was tested.

The antifungal activity of the biosynthesized AgNPs was investigated against planktonic cells of *C*. *albicans* ATCC 10231 in various concentrations (0, 2.5, 5.0, 10.0, 20.0, and 40.0 mg/L). It was found that the extract of *V*. *vinifera* canes alone did not inhibit the growth of the planktonic cells. On the contrary, at maximal tested concentration of the *V*. *vinifera* extract, an increase up to 130 rel. % of cell culture was observed.

**Fig 4:**
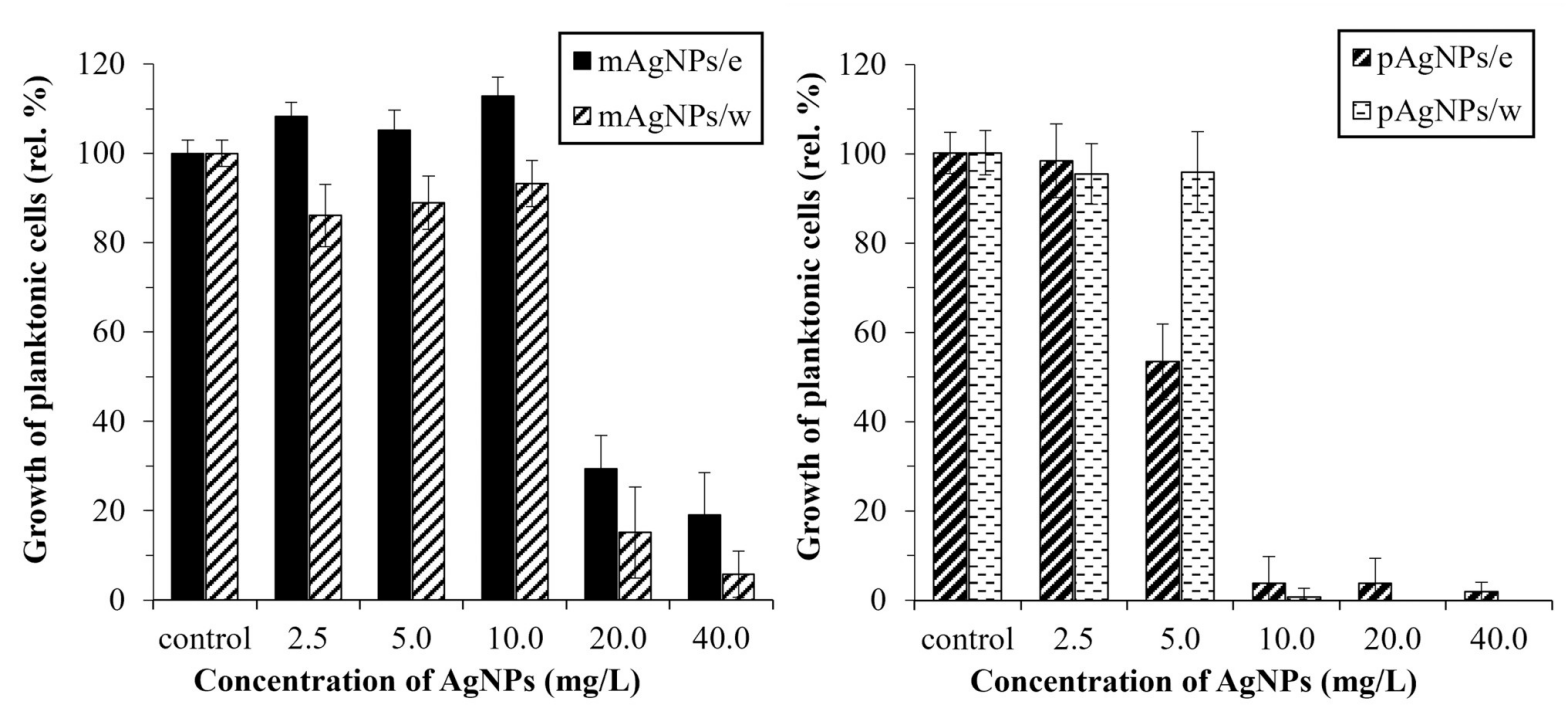
Morphology-dependent antimicrobial activity of AgNPs biosynthesized using *V*. *vinifera* cane extract against planktonic cells of *C*. *albicans*. (mAgNPs–monodisperse AgNPs; pAgNPs–polydisperse AgNPs; /e–*V*. *vinifera* extract; /w–ultrapure water as medium).

Both biosynthesized silver NPs displayed significant inhibition activity against *C*. *albicans*. Monodisperse nanoparticles significantly inhibited the growth of the microorganism at concentration 20 mg/L and higher (Fig 4). Using mAgNPs, MIC_80_ (20 mg/L) was observed only using mAgNPs in water as medium. Polydisperse AgNPs showed higher antifungal activity and even 10 mg/L of pNPs inhibited the growth of the microorganism by more than 80 rel. %, in both media. Polydisperse AgNPs were effective even using 10 mg/L (inhibition at least 96 rel. %), while using 20 mg/L of pAgNPs/w completely inhibited the growth of yeast (MIC). Wypij, Czarnecka [36] reported even higher MIC (32 mg/L) than in our study. The authors tested activity of polydisperse biogenic AgNPs (size interval 5–20 nm). A similar effect was observed in Jain, Arora [37] where spherical AgNPs in the size range 7–20 nm showed MIC of yeast *C*. *albicans* 25 mg/L.

Our results indicate that the presence of the *V*. *vinifera* extract decreases the antimicrobial activity of the NPs which correlates with our observation that *V*. *vinifera* cane extract enhances the growth of *Candida* planktonic cells and thus our hypothesis of synergistic interactions was not confirmed against planktonic cells of *C*. *albicans*. Increased activity of isolated AgNPs was observed in both nanoparticle morphologies, even though the reaction yield was up to 91–98% and therefore the extract-containing dispersions of AgNPs (AgNPs/e) contained minor amount of unreacted silver ions. However, the activity of free Ag^+^ ions is known to immediately decrease due to reduction by the presence of chlorides in the media or buffer.

Regarding the antimicrobial efficiency of nanoparticles with different morphology, the results clearly showed that polydisperse AgNPs showed higher activity, even though the smaller particles contained in monodisperse AgNPs are usually reported to have higher antimicrobial activity [38, 39]. This finding confirms that the size of the nanoparticles is not the only factor that affects the antimicrobial effect. We could explain the higher activity of polydisperse nanoparticles by a broader spectrum of possible mechanisms of different sizes and shapes of NPs in comparison with a sample containing NPs of high circularity and a narrow size range. Morphology-dependent antimicrobial activity of metal nanoparticles has been already recorded. As the main cause of different activity of NPs of different shapes and sizes is considered the rate of silver ions release from the surface of nanoparticles [40, 41].

**Fig 5:**
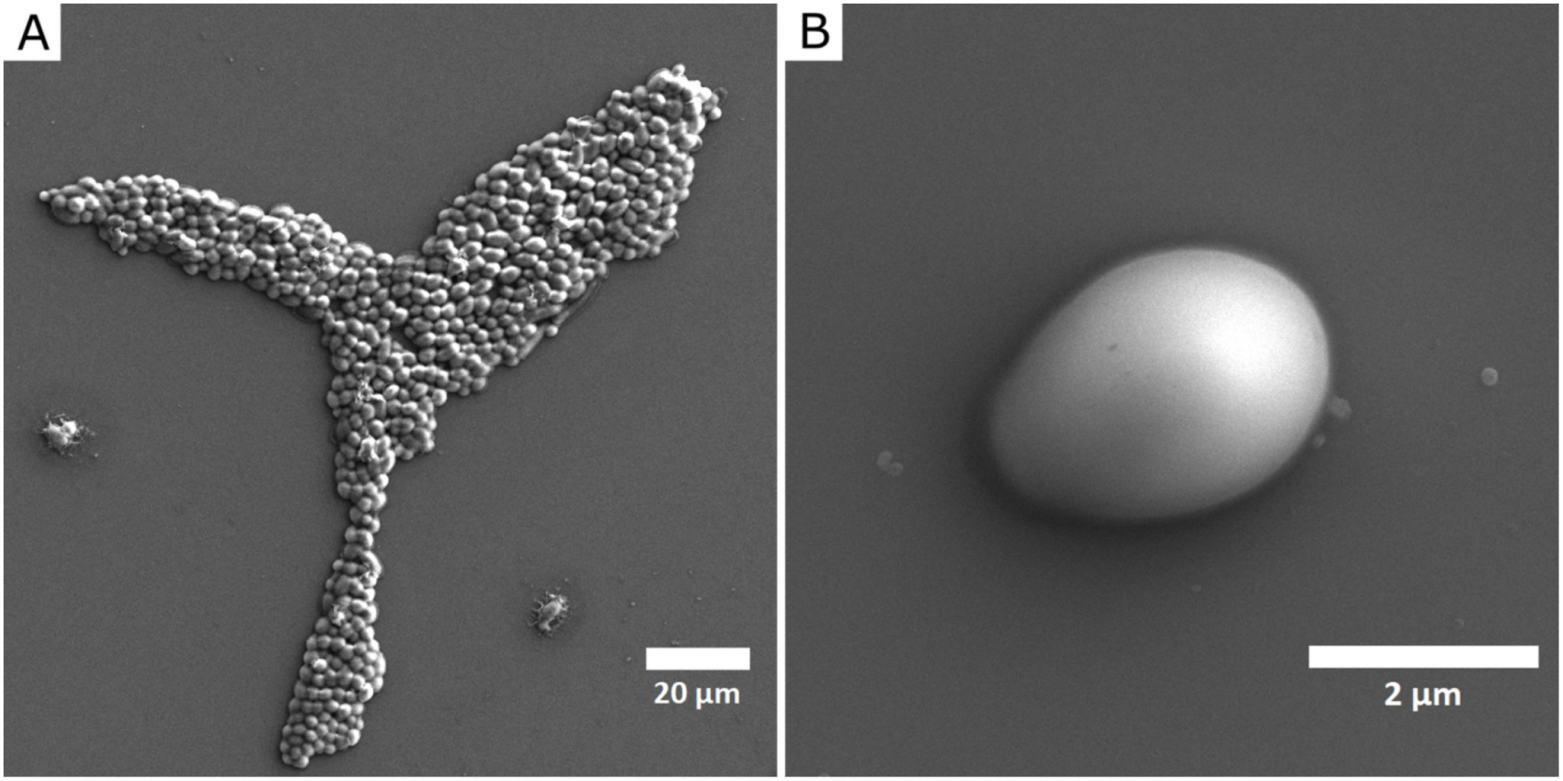
SEM images of *C*. *albicans* ATCC 10231 planktonic cells (without NPs). (A) scale 20 μm; (B) scale 2 μm.

**Fig 6:**
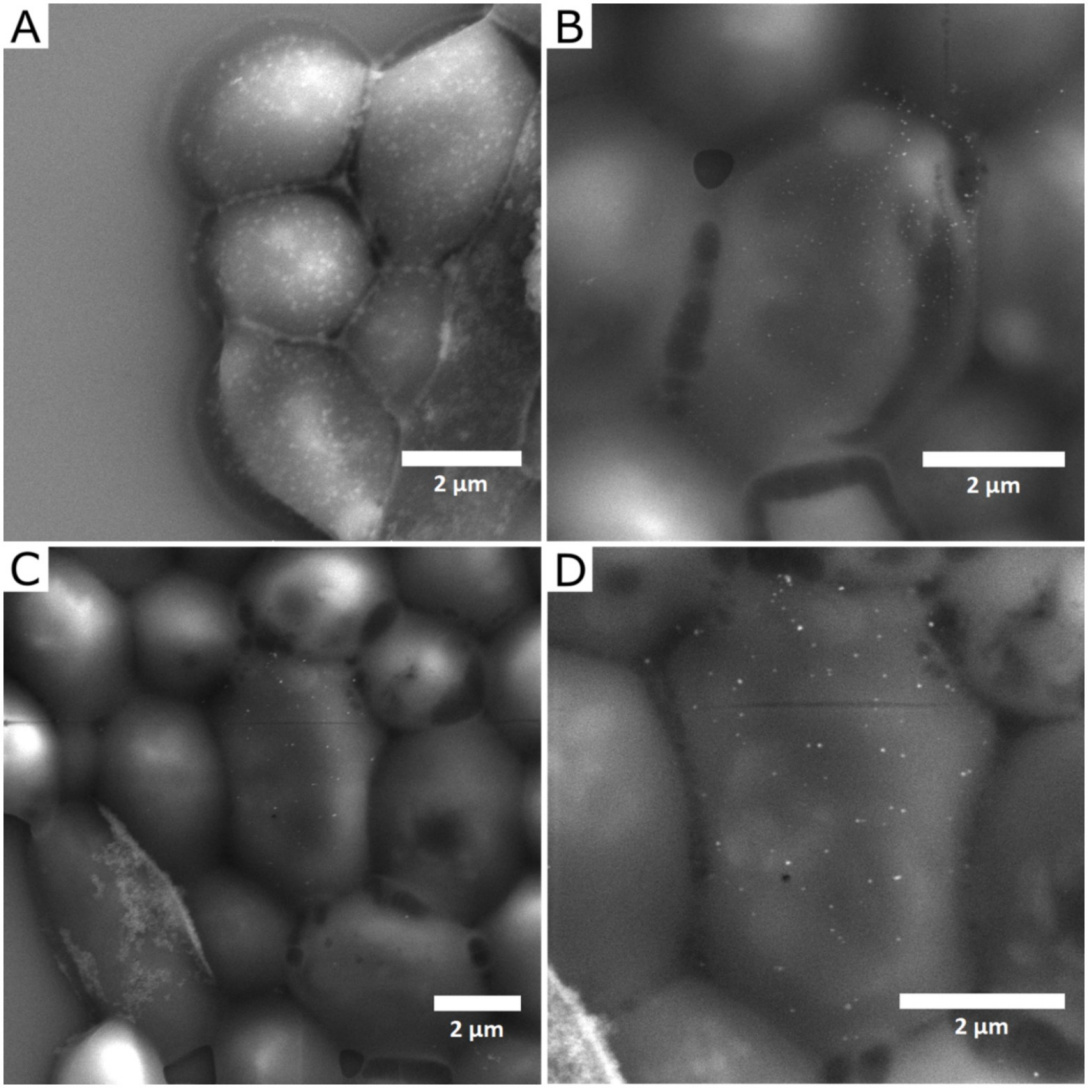
SEM images of *C*. *albicans* cells treated for 24 h with 5 mg/L of monodisperse AgNPs biosynthesized using *V*. *vinifera* extract (mAgNPs). (**A** and **B**) cells treated with mAgNPs in extract; (**C** and **D**) cells treated with mAgNPs in ultrapure water.

**Fig 7:**
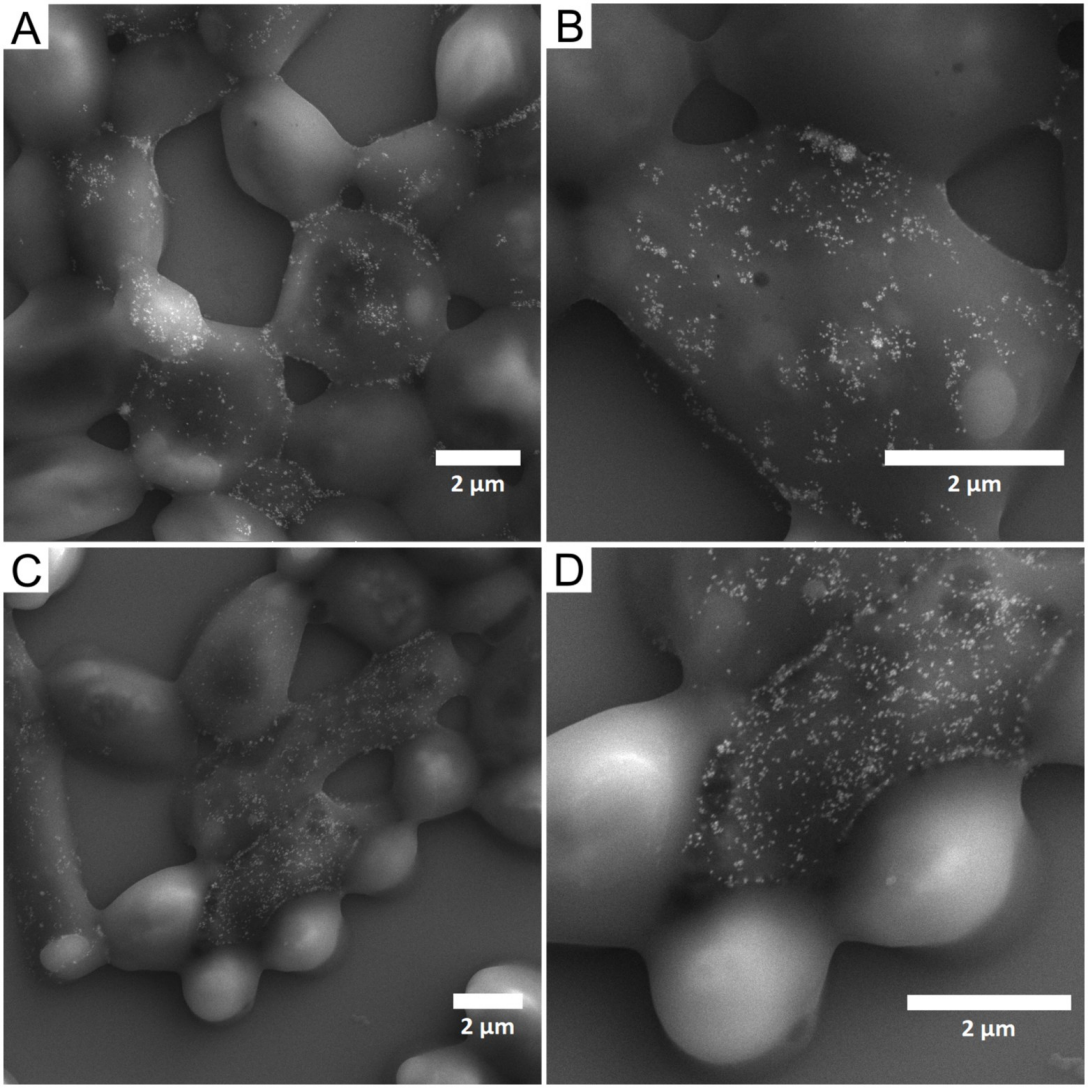
SEM images of *C*. *albicans* cells treated with 5 mg/L of polydisperse AgNPs biosynthesized using *V*. *vinifera* extract (pAgNPs). (**A** and **B**) cells treated with pAgNPs in extract; (**C** and **D**) cells treated with pAgNPs in ultrapure water.

Interactions of NPs and *C*. *albicans* cells were visualized by SEM (images of untreated, monodisperse AgNPs treated and polydisperse AgNPs treated cells of *C*. *albicans* are shown in Figs 5–7, respectively). The mechanism of action of silver NPs includes the release of metal ions, oxidative stress associated with ROS production, and various nonoxidative mechanisms, including direct interactions with the cell wall (and/or cell membrane) or intracellular penetration [12]. The adsorption of NPs to the cell wall of the microorganism can lead to its disruption, which affects the integrity and permeability of the membrane [12, 36]. The cell wall and cell membrane maintain cell integrity and stability and play a crucial role in the pathogenicity and virulence of pathogenic fungi [42]. The degradation of the cell wall allows the NPs and silver ions to enter the cell interior and cause subsequent damage such as inhibition of ATP production and DNA replication [43]. In the present study, the interaction of nanoparticles with the *C*. *albicans* cell surface was studied at concentration of 5 mg/L of AgNPs (the lowest concentration at which the first significant decrease in planktonic cell was observed: specifically using pAgNPs/e, see Fig 4). The applications of both mAgNPs and pAgNPs resulted in visible interactions with the cell wall of the microorganism. While untreated cells (Fig 5) had a smooth and regular surface and retained their typical oval shape, visible damage of the cell surface was observed for treated cells and NPs were found directly adhered to cells in all cases (Figs 6 and 7).

Cells after treatment with mAgNPs/e had NPs visibly adhered to the surface (Fig 6), but the morphology of their surface was not as fundamentally affected as the use of mAgNPs isolated in water and polydisperse AgNPs. This is correlated with the limited inhibition of cell growth when 5 mg/L of mAgNPs/e did not inhibit the cell growth at all, unlike other samples of NPs. The treated cells were found to change the typical oval shape and to shrink. Although the treated cells were clearly disrupted (eg. Fig 7A), the sample obviously contained cells with a smooth surface resembling untreated cell. This indicates that after the adhesion of the nanoparticles to the cell surface, a lot of viable cells remained in the sample, so the effect of the NPs in used concentration is only fungistatic.

Our observations are in agreement with previous studies of *C*. *albicans* cell-NPs biointerface. Radhakrishnan, Mudiam [44] reported the activity of chemically synthesized spherical silver NPs (10–30 nm) against *C*. *albicans*. They found MIC_90_ (minimum concentration required to inhibit the growth of 90% of organisms) 40 mg/L of NPs and subsequently studied how this concentration of NPs affects the cell envelopes. Similar as in the present study, obvious changes in cell wall surface were detected, even that the AgNPs concentration used for cell treatment was eightfold higher.

### Antibiofilm activity of AgNPs

Biofilm formation provides the pathogenic microorganism with an extraordinary ability to invade the host, adapt and modify its immediate environment. Thus, the microbial population in a biofilm can withstand adverse environmental conditions and demonstrate dramatically reduced susceptibility to antibiotics, detergents, and host immune responses [45]. Biofilm prevents the action of antibacterial agents in a number of ways, such as difficult diffusion of antimicrobials through the extracellular matrix, altered phenotype, slow cell growth, or the presence of persistent cells [46]. Consequently, a large part of antimicrobial research is focused on antibiofilm approaches. Silver or copper NPs were found to be able to prevent biofilm formation, and are therefore proposed as advantageous as coatings for implants to fight implant-associated infections [47]. Recently, AgNPs have proven its efficacy against *C*. *albicans* biofilms [48, 49].

In the present study, the antibiofilm potential of biosynthesized silver NPs (0, 2.5, 5.0, 10.0, and 20.0 mg/L) of two different morphologies was studied. The metabolic activity of treated and untreated *C*. *albicans* biofilm cells was monitored using a resazurin assay.

**Fig 8:**
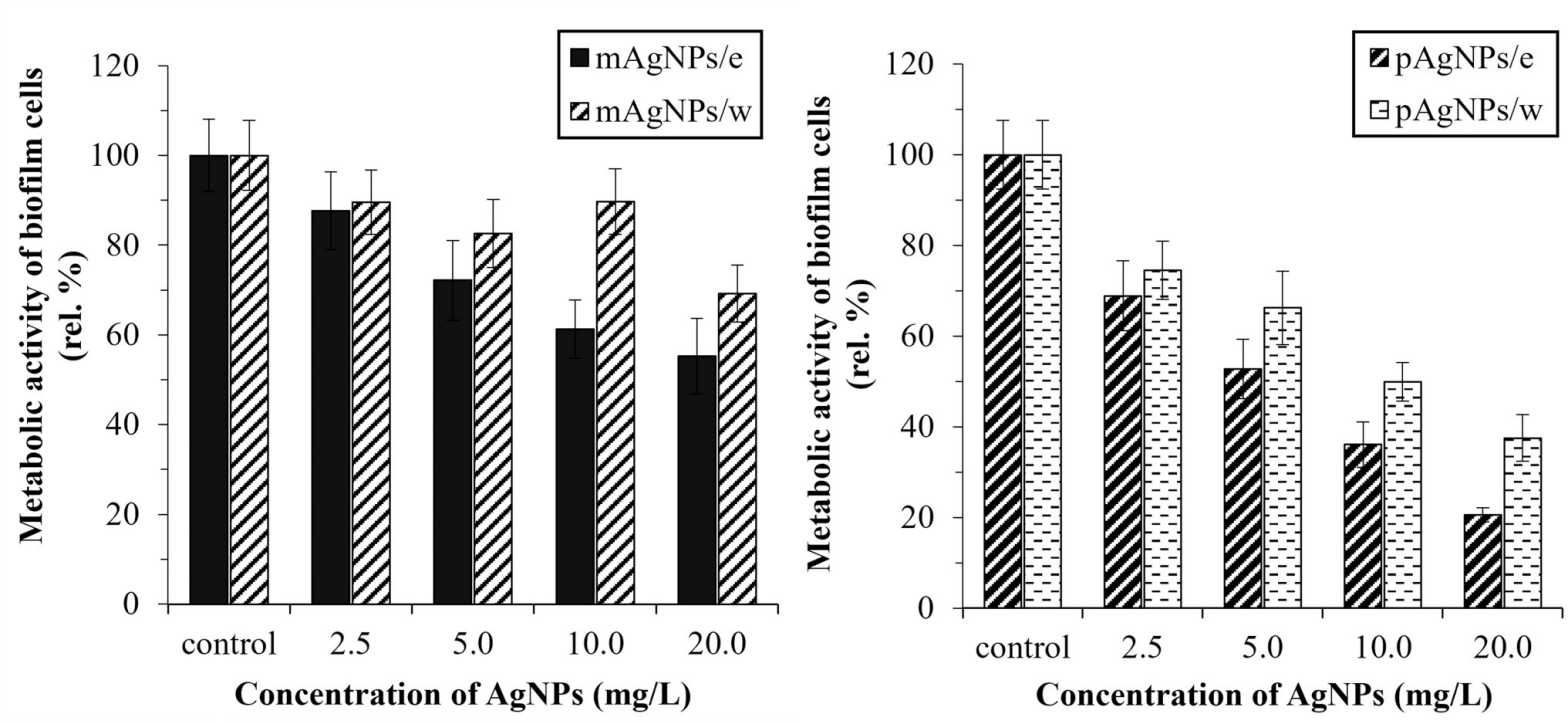
Morphology-dependent antibiofilm activity of AgNPs biosynthesized using *V*. *vinifera* cane extract against biofilm cells of *C*. *albicans*. (mAgNPs–monodisperse AgNPs; pAgNPs–polydisperse AgNPs; /e–*V*. *vinifera* extract; /w–ultrapure water as medium); measured using resazurin assay.

*V*. *vinifera* cane extract has been found to inhibit the metabolic activity of biofilm cells, which differs from our observation of the effects on planktonic cells. The highest concentration of the extract (2% (v/v)) inhibited the metabolic activity by 32 rel. % in comparison to control. Our results are in agreement with the results of Rollová, Gharwalova [22], where the inhibitory effects of *V*. *vinifera* extract on the metabolic activity of *E*. *coli* and *Citrobacter freundii* biofilms were demonstrated. Interestingly, the nanoparticles had an increased inhibitory effect in combination with *V*. *vinifera* extract as a medium (compared to those isolated in ultrapure water), therefore confirming our hypothesis of synergistic activity of NPs and plant extract for *C*. *albicans* biofilm cells (Fig 8).

Polydisperse AgNPs were more effective in inhibiting the activity of biofilm cells, similarly to their effect on planktonic cells. The highest decrease in metabolic activity (80 rel. %) was observed at concentration 20 mg/L pAgNPs/e. The monodisperse AgNPs (20 mg/L) decreased the metabolic activity of biofilm cells by less than 50 rel. %.

## Conclusion

In this work, we have achieved biosynthesis of stable AgNPs of different morphologies and subsequent different antimicrobial and antibiofilm properties in a controlled manner. The results confirm the differences in antifungal and antibiofilm activity of AgNPs of various dispersity (mono- and polydisperse). Larger, polydisperse AgNPs showed to be more effective against both planktonic and biofilm cells, than smaller monodisperse AgNPs. The hypothesis of synergistic interaction of biologically active molecules from *V*. *vinifera* extracts and AgNPs was proved for the antibiofilm assays.

## Supporting information

S1 Fig UV-Vis confirmation of successful isolation of biosynthesized AgNPs into ultrapure water. A–isolation of monodisperse AgNPs; B–isolation of polydisperse AgNPs (mAgNPs–monodisperse AgNPs; pAgNPs–polydisperse AgNPs; /e–*V*. *vinifera* extract; /w–ultrapure water as medium).
(TIF)
