# Supplementary figures and images for: Antibiofilm activity of silver nanoparticles biosynthesized using viticultural waste
Source: PLoS One. 2022 Aug 10;17(8):e0272844. doi: 10.1371/journal.pone.0272844 (PMC9365141; doi:10.1371/journal.pone.0272844)

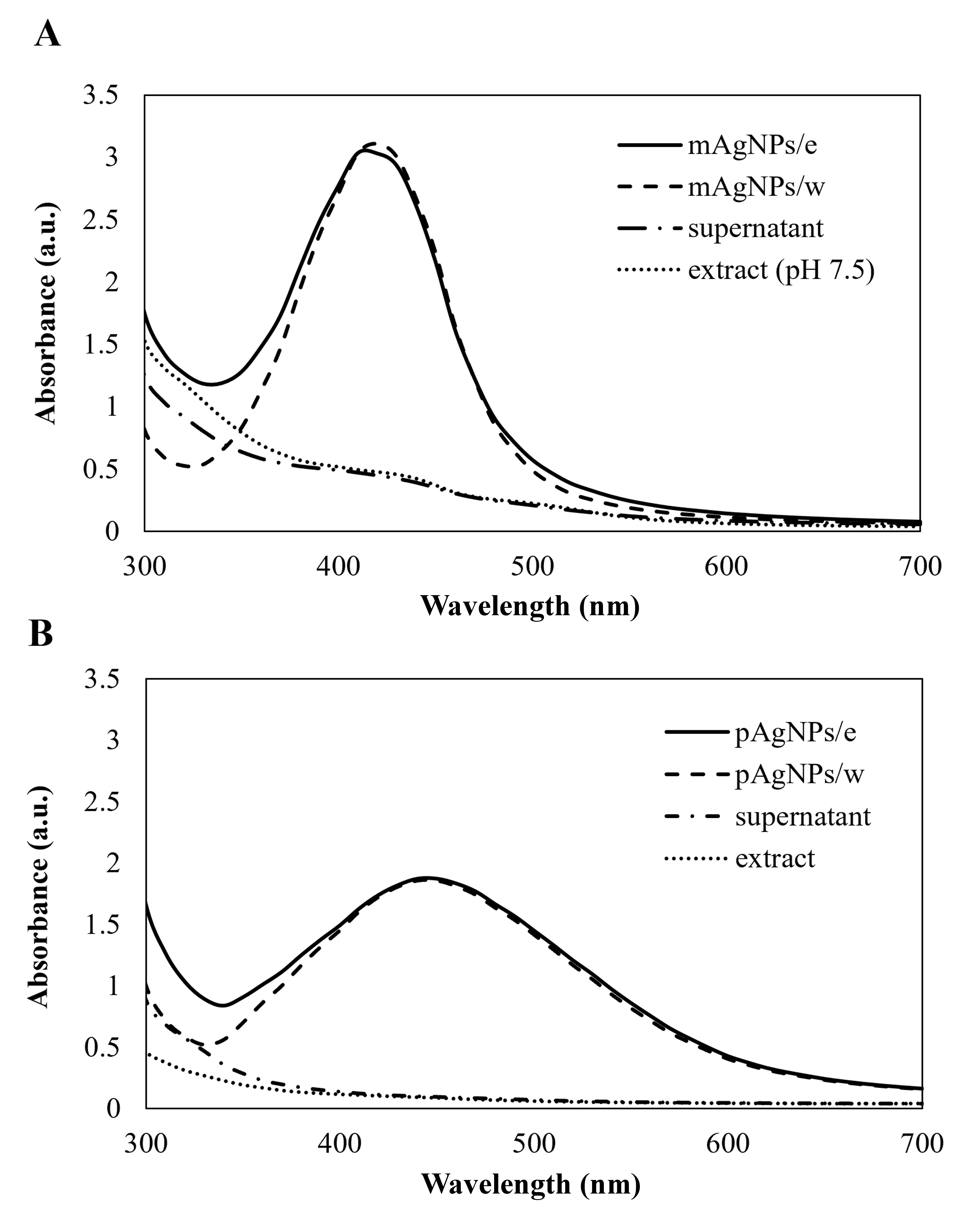

Supplement: S1 Fig — A–isolation of monodisperse AgNPs; B–isolation of polydisperse AgNPs (mAgNPs–monodisperse AgNPs; pAgNPs–polydisperse AgNPs; /e–V. vinifera extract; /w–ultrapure water as medium). (TIF) [file pone.0272844.s001.tif]
